# Updating Our View of Organelle Genome Nucleotide Landscape

**DOI:** 10.3389/fgene.2012.00175

**Published:** 2012-09-11

**Authors:** David Roy Smith

**Affiliations:** ^1^Department of Botany, Canadian Institute for Advanced Research, University of British ColumbiaVancouver, British Columbia, Canada

**Keywords:** *Coccomyxa*, GC content, mitochondrial DNA, plastid DNA, *Polytomella*, *Selaginella*, RNA editing

## Abstract

Organelle genomes show remarkable variation in architecture and coding content, yet their nucleotide composition is relatively unvarying across the eukaryotic domain, with most having a high adenine and thymine (AT) content. Recent studies, however, have uncovered guanine and cytosine (GC)-rich mitochondrial and plastid genomes. These sequences come from a small but eclectic list of species, including certain green plants and animals. Here, I review GC-rich organelle DNAs and the insights they have provided into the evolution of nucleotide landscape. I emphasize that GC-biased mitochondrial and plastid DNAs are more widespread than once thought, sometimes occurring together in the same species, and suggest that the forces biasing their nucleotide content can differ both among and within lineages, and may be associated with specific genome architectural features and life history traits.

## Introduction

Mitochondria and plastids are the products of ancient endosymbiotic events, involving a proteobacterium and a cyanobacterium, respectively (Lang et al., 1999; Palmer, 2003). Mitochondria arrived early and probably existed in the common ancestor of all eukaryotes (Gray et al., 1999). Plastids came later, first arising in the Archaeplastida (Plantae), and then being passed on laterally to diverse lineages through eukaryote–eukaryote endosymbioses (Archibald, 2009; Keeling, 2010). The genomes within contemporary mitochondria and plastids have been fashioned through coexistence and coevolution with their eukaryotic hosts, and in many instances have acquired bizarre and complex architectures (Palmer, 1985; Gray et al., 2004; Green, 2011).

Organelle DNAs boast an impressive, and often puzzling, array of sizes (<10 to >1000 kb), conformations (circular or linear), chromosome numbers (monomeric to highly fragmented), compactnesses (<10 to >90% non-coding DNA), and gene repertoires (<5 to >250 genes). Moreover, many organelle genomes use a non-standard genetic code (Jukes and Osawa, 1993), and some employ complicated editing systems that alter the sequences of RNA transcripts (Covello and Gray, 1989; Simpson and Thiemann, 1995). One feature of organelle DNA that has proven to be relatively constant across lineages is its nucleotide composition. Almost all completely sequenced mitochondrial and plastid DNAs (mtDNAs and ptDNAs) have a high adenine and thymine (AT) content (Kusumi and Tachida, 2005; Min and Hickey, 2007). Various hypotheses have tried to explain this AT bias, but the topic remains poorly understood.

Recently, it was shown that guanine and cytosine (GC)-rich organelle DNAs do exist (Tsuji et al., 2007; Smith and Lee, 2008; Hecht et al., 2011). These genomes come from a small but diverse group of species, including various green plants and animals, and sometimes have linear conformations or undergo large amounts of post-transcriptional editing. Unraveling the mechanism responsible for their GC enrichment may help explain the near-ubiquity of AT-rich mitochondrial and plastid genomes throughout the eukaryotic domain, and could give insights into other aspects organelle genome architecture, such as the origins of RNA editing. The existence of GC-rich organelle genomes, however, is poorly chronicled in the scientific literature, even though these sequences could impact how we use organelle DNA for studying molecular evolution (Foster and Hickey, 1999).

This review showcases GC-biased organelle genomes and the species in which they are found. GC enrichment is discussed in context to mutation, recombination, population genetics, and genome architecture. It is emphasized that GC-rich mtDNAs and ptDNAs are more common than once thought – occasionally occurring together in the same species – and that the processes promoting GC enrichment can differ within and among lineages.

## The Near-Ubiquity of AT-Rich Organelle Genomes

**Figure 1:**
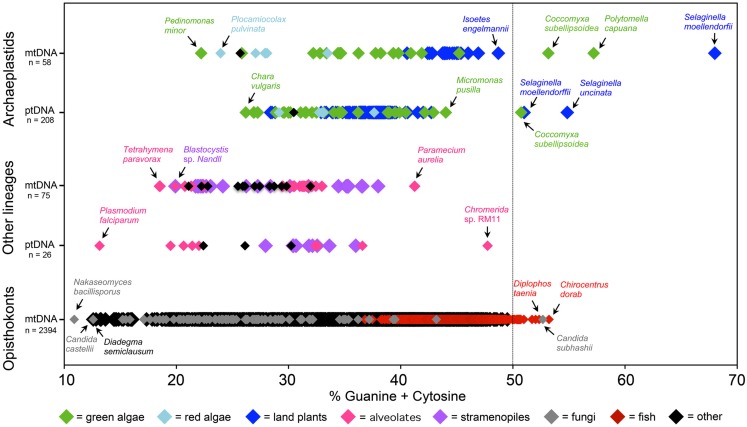
**Nucleotide composition continuum of completely sequenced mitochondrial DNA (mtDNA) and plastid DNA (ptDNA) sequences**. Most of the complete organelle genome sequences deposited in GenBank have a GC content below 50%, with the exception of those from certain green algae, lycophytes, fish, and fungi. The number of genome sequences (*n*) within each group is shown beside the *y*-axis. Mitochondrial and plastid genome sequences were downloaded from GenBank on January 1, 2012.

The sequencing of large numbers of organelle genomes from diverse lineages has revealed an almost universal AT bias in mtDNAs and ptDNAs across the eukaryotic domain (Figure 1). Of the ∼2,500 mitochondrial and plastid genomes that have been sequenced, as of January 1, 2012, most have an AT content above 50% (average ≈65%; Figure 1). Convergent evolution to AT richness is found in other types of organelle-located DNAs, such as mitochondrial plasmids (Handa, 2008), nucleomorph genomes (Moore and Archibald, 2009), and the genomes of mitochondrial viruses (Wu et al., 2010). Moreover, the genomes of bacterial and eukaryotic endosymbionts and intracellular parasites tend to have higher AT compositions than those of their free-living close relatives (Pallen and Wren, 2007; Nowack et al., 2008; McCutcheon and Moran, 2010).

Many processes can influence nucleotide landscape, including mutation, recombination, random genetic drift, and selection (Lynch, 2007; Charlesworth and Charlesworth, 2010). The net effect of these processes ultimately determines the equilibrium nucleotide composition of a genome. The origins of AT richness within mtDNAs and ptDNAs are thought to reflect the endosymbiotic history of these genomes, their location within the cell, the unique population-genetic features that define organelles, and selection for metabolic and translational efficiency.

The massive shedding of genes that characterized early mtDNA and ptDNA evolution resulted, at least for some lineages, in the loss of key DNA repair proteins and, consequently, diminished nucleotide repair capacities within organelles (Kleine et al., 2009; Bendich, 2010, but see Liu and Demple, 2010). Organelle DNAs are typically uniparentally inherited, non-recombining, and can experience severe bottlenecks during transmission, which implies that they are inefficient at purging deleterious mutations from their populations (Muller, 1964; Rand, 2001; but see Piganeau et al., 2004). Organelle genomes undergo multiple rounds of replication per cell division (Birky, 2001), predisposing them to replication errors, and they are housed in energy-producing compartments where high concentrations of reactive oxygen species promote GC→AT mutations through the deamination of cytosine and the oxidative conversion of guanine to 8-oxo-guanine (Martin, 1995; Asada, 2006; Murphy, 2009; Shokolenko et al., 2009). Together, these points suggest that organelle DNAs inhabit a highly mutagenic environment, where DNA repair is inefficient, and the mutational spectrum is skewed toward AT. As one might expect, many species, including most metazoans, appear to have high organelle DNA mutation rates (Lynch et al., 2006). There are some species, however, for which the organelle DNA mutation rate is estimated to be low (e.g., most angiosperms), yet their organelle genomes are still AT-rich (Drouin et al., 2008).

In addition to a genome-wide AT bias, mtDNAs and ptDNAs can exhibit regional and strand-specific nucleotide biases (Gibson, 2005; Kusumi and Tachida, 2005). The mutational consequences of organelle genome replication can give rise to AC vs. GT inequities because the DNA strand that spends more time in the mutationally vulnerable single-stranded state is prone to C→T and A→G transitions (Ames et al., 1995; Frank and Lobry, 1999; Faith and Pollock, 2003); but this does not impact the overall AT composition as the G’s and T’s of one strand are complemented by A’s and C’s on the other strand.

Natural selection is thought to have contributed to the high AT content of mitochondrial and plastid genomes. Selection for translational efficiency and accuracy is believed to have shaped the nucleotide composition of codons in organelle genes, in some cases enriching the thymine content of synonymous sites (Morton, 1998). Others have argued that AT richness is an adaptation for metabolic efficiency, noting the increased energetic costs of producing C vs. T and G vs. A and the varying abundance of A/T vs. G/C nucleotides during organelle DNA synthesis (Jukes and Bhushan, 1986; Wolfe, 1991; Rocha and Danchin, 2002).

Thus, a multitude of forces have likely helped generate the near-universal AT bias of organelle DNAs. The discovery of organelle genomes with a high GC content has provided an important point of comparison from which to better understand these forces.

## Taxa with GC-Rich Organelle Genomes

**Figure 2:**
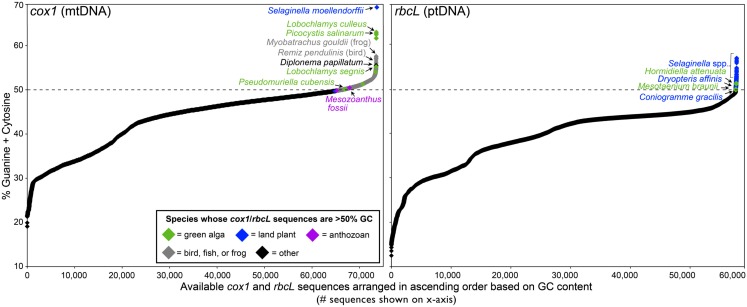
**Nucleotide composition continuum of the available *cox1* and *rbcL* sequences from eukaryotic organelle genomes**. The *cox1* gene, which is located in the mitochondrial genome of all studied eukaryotes, encodes the protein cytochrome c oxidase subunit I. The *rbcL* gene, which is found in the ptDNA of most plastid-bearing eukaryotes, encodes the large subunit of Rubsico. The nucleotide content of *cox1* and *rbcL* are good predictors of the overall mtDNA and ptDNA nucleotide composition, respectively (Min and Hickey, 2007; Smith, 2009). Complete and partial *cox1* and *rbcL* sequences (minimum length = 400 nt) were downloaded from GenBank on January 1, 2012. Given the huge number of bilaterian *cox1* sequences (>300,000), the chart only shows those for species from the Actinopterygii, Archosauria, and Amphibia – the bilaterians known to have *cox1* sequences that can exceed 50% GC.

**Figure 3:**
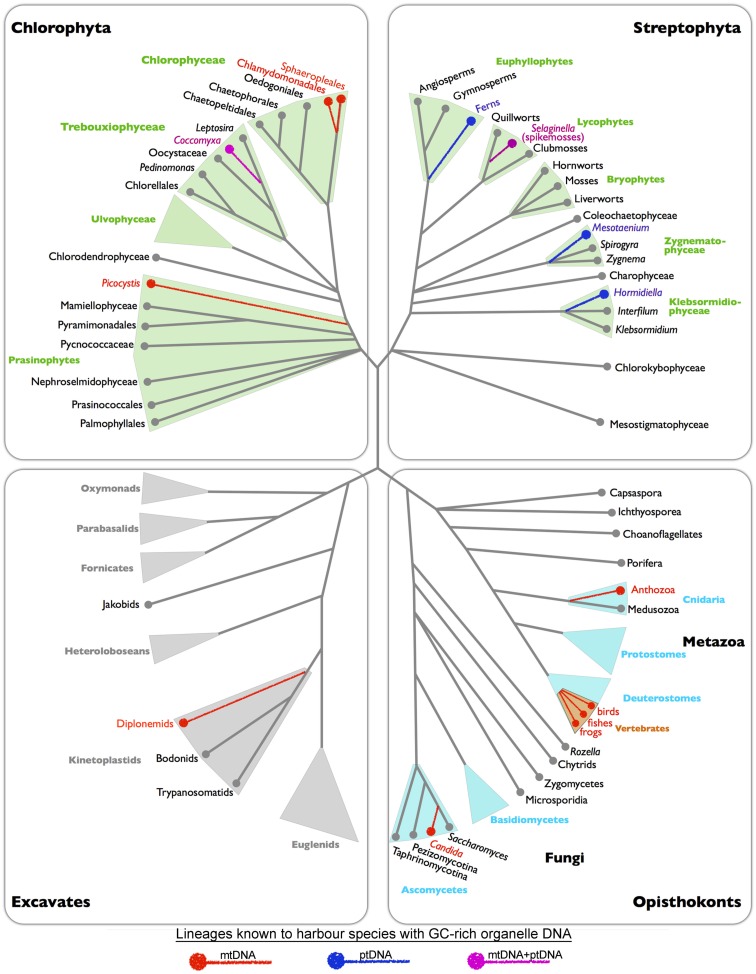
**Tree showing the eukaryotic lineages that have species with GC-rich organelle genomes**. Colored branches represent lineages that are known to contain (based on complete organelle genome sequence data) or predicted to contain (based on *cox1*/*rbcL* sequences) species with GC-rich organelle genomes (red = mtDNA, blue = ptDNA, pink = mtDNA and ptDNA). Branching order based on published phylogenetic analyses.

There are more than 40 complete organelle genome sequences in GenBank with GC contents exceeding 50% (Figure 1). These genomes come from various fish, green algae, and land plants as well as a fungus (Figure 1; Table 1). Moreover, the nucleotide composition of organelle genes, like those encoding the mitochondrial protein cytochrome c oxidase subunit I (*cox1*) and the large subunit of the plastid protein Rubisco (*rbcL*), have proven to be good predictors of overall organelle DNA nucleotide content (Min and Hickey, 2007; Clare et al., 2008; Smith, 2009). Analyses of *cox1* and *rbcL* have revealed other lineages with GC-rich organelle genomes (Kerr et al., 2007; Borza et al., 2009; Figure 2; Table 1). The taxonomic groups containing (or predicted to contain) species with GC-rich organelle DNA are listed below and highlighted in Figures 1–3 and Table 1.

**Table 1:** **Examples of GC-rich organelle genomes and the species that harbor them**.

	Taxonomy	GC_TOT_	GC_1_	GC_2_	GC_3_	GC_NC_	GC-bias mt + pt^2^	Other taxa^3^	Genomic architecture	Organismal features	GenBank accession
**MtDNAs**											
*Myobatrachus gouldii*^1^	Frog (turtle frog)	∼55	59.3	44.4	69.5	N/A	–	Yes	N/A	Small burrowing species found in sandy soil throughout Western Australia.	HQ584074-5 AY948768
*Candida subhashii* strain CBS 10753	Fungus (yeast)	52.7	53.2	38.7	66.4	54.4	–	No	30 kb compact intron-less linear genome with inverted-repeat telomeres (Fricova et al., 2010).	Human pathogen. First isolated from a case of fungal peritonitis.	NC_014337
*Chirocentrus dorab*	Fish (wolf herring)	53.2	58.7	47.7	53.7	39.3	–	Yes	16 kb compact intron-less circular-mapping genome (Ishiguro et al., 2005).	Marine; brackish. Distribution Indo-Pacific. Observed in warm coastal waters.	NC_006913 AP006229
*Coccomyxa subellipsoidea* C-169	Green alga (trebouxiophyte)	53.2	51.4	40.8	59.8	55.7	Yes	Yes	65 kb, circular-mapping genome with moderate amount of non-coding DNA. Similar repeat elements in mtDNA and ptDNA (Smith et al., 2011).	Free-living, unicellular species, isolated in Marble Point Antarctica.	HQ874522
*Diplonema papillatum* ATCC 50162^1^	Protist (euglenozoan)	∼55	60.9	53.0	53.2	∼54	–	No	Multipartite genome comprised of circular-mapping chromosomes. Highly fragmented coding regions. U-insertion RNA editing (Vlcek et al., 2011).	Free-living, unicellular marine flagellate, isolated from the surface of eelgrass in New Hampshire.	HQ288819-33 EU123536-7
*Lobochlamys culleus* SAG19.72^1^	Green alga (chlorophycean)	∼60	53.4	40.5	93.0	∼59	No	Yes	Multipartite genome comprised of linear chromosomes with overlapping homologies. Repeat dense (Borza et al., 2009).	Free-living, unicellular freshwater biflagellate. Isolated from pond in Florida, USA.	AF529310-6 FJ393025-57
*Mesozoanthus fossii*^1^	Coral (zoanthid)	∼55	50.1	40.9	64.2	N/A	–	Yes	N/A	Observed in fjords from Northern to Central Patagonia.	EF672653-5 EF687821-3
*Isoetes lacustris*^1^	Land plant (lycophyte)	∼50	59.8	63.8	43.3	∼58	No	Yes	∼60 kb genome, comprising a complex network of recombinogenic mtDNA molecules. High levels of C-to-U RNA editing. Intron rich, but relatively compact (Grewe et al., 2009)^4^.	Boreal quillwort observed in Europe and North America. Grows on the bottom of ponds.	AM261455-6 Y17812-4 X92736
*Picocystis salinarum* CCMP 1897^1^	Green alga (prasinophyte)	∼60	55.4	41.1	91.7	N/A	No	N/A	N/A	Unicellular picoplankton, isolated from a saline pond in San Francisco Bay.	AB491634
*Polytomella capuana* SAG 63-5	Green alga (chlorophycean)	57.2	52.2	41.3	76.0	61.0	N/A	No	13 kb highly reduced, linear genome with inverted-repeat telomeres (Smith and Lee, 2008).	Free-living, non-photosynthetic unicellular freshwater flagellate, isolated from ditch in Italy.	NC_010357
*Selaginella moellendorffii*	Land plant (lycophyte)	68.1	64.2	60.2	61.5	68.9	Yes	Yes	Genome comprised of a complex network of recombinogenic mtDNA molecules. Repeat and intron dense. Unprecedented levels of C-to-U RNA editing (Hecht et al., 2011).	Seedless vascular plant. Model species, often used for cultivation.	GQ246802-8 JF338143-7
*Remiz pendulinus*^1^	Bird (penduline tit)	∼55	58.6	42.2	65.4	N/A	–	Yes	N/A	Tiny passerine observed in various regions throughout Eurasia.	GU572078-9 AY228081
**PtDNAs**											
*Cheiropleuria integrifolia*^1^	Land plant (fern)	∼50	59.6	45.7	45.9	∼50	N/A	Yes	N/A	Terrestrial fern of moderate size. Collected in Japan, Kagoshima prefecture (Ebihara et al., 2010).	AB042569 EU328229
*Coccomyxa subellipsoidea* C-169	Green alga (trebouxiophyte)	50.7	56.1	43.6	50.1	51.0	Yes	Yes	176 kb intron-poor circular-mapping genome. Similar repeat elements in mtDNA and ptDNA (Smith et al., 2011).	Free-living, unicellular species, isolated in Marble Point Antarctica.	NC_015084
*Coniogramme gracilis* Ogata^1^	Land plant (fern)	∼50	59.3	43.8	48.7	N/A	N/A	Yes	N/A	Narrow-leaf bamboo fern. Collected in Japan, Kagoshima prefecture (Ebihara et al., 2010).	AB574810
*Hormidiella attenuata* strain M2214^1^	Green alga (klebsormidiophyte)	∼51	58.4	42.5	52.9	N/A	N/A	Yes	N/A	Freshwater species, forming multicellular, non-branching filaments.	HQ613235
*Mesotaenium braunii*^1^	Green alga (zygnemophyte)	∼51	58.5	43.1	51.5	N/A	N/A	No	N/A	Free-living, unicellular freshwater alga. Isolated from Eifel, Germany.	FM992358 FM992569
*Selaginella uncinata*	Land plant (lycophyte)	54.8	58.8	54.7	49.3	54.9	Yes	Yes	144 kb intron-poor circular-mapping genome. Reduced tRNA-coding content. High levels of C-to-U RNA editing (Tsuji et al., 2007).	Seedless vascular plant. Model species, often used for cultivation.	AB197035

*Percentage of guanine and cytosine of entire genome (GC_TOT_), first-, second-, and third-position codon sites (GC_1_, GC_2_, GC_3_, respectively), non-coding regions (GC_NC_). Data not available (N/A)*.

*^1^Statistics based on partial genome sequence data (e.g., *rbcL* or *cox1*)*.

*^2^Reports if a GC bias is present in both the mitochondrial and plastid genomes (only applicable to plastid-bearing species)*.

*^3^Reports if GC-rich organelle DNA has been observed in other members of the given lineage*.

*^4^Genomic architecture based on data for *Isoetes engelmannii**.

### Green algae

Some of the highest organelle genome GC contents come from green algae. The chlorophycean *Polytomella capuana*, a non-photosynthetic unicell closely related to the model organism *Chlamydomonas reinhardtii*, has an mtDNA GC content of 57% (Smith and Lee, 2008). All other investigated *Polytomella* species, however, have AT-rich mtDNAs (Smith et al., 2010). Partial mitochondrial genome sequences suggest that the freshwater biflagellates *Oogamochlamys gigantea*, *Lobochlamys segnis*, and *Lobochlamys culleus*, which are also close relatives of *C. reinhardtii*, have mtDNA GC compositions of approximately 50, 55, and 60%, respectively (Borza et al., 2009). The polar trebouxiophyte *Coccomyxa subellipsoidea* C-169 has a GC-bias in both its mitochondrial and plastid compartments (53 and 51% GC, respectively), and organelle gene sequencing indicate that *Coccomyxa chodatii* and *Coccomyxa rayssiae* have GC-rich organelle DNAs as well (Smith et al., 2011). The picoplankton *Picocystis salinarum*, a deep-branching prasinophyte, appears to have an mtDNA GC content exceeding 60%. And *rbcL* sequences imply that there are GC-enriched plastid genomes in select members of the charophyte genera *Mesotaenium* and *Hormidiella* (Gontcharov and Melkonian, 2010; Rindi et al., 2011).

### Land plants

The highest recorded GC content in an mtDNA (68%) and a ptDNA (55%) belong to the seedless vascular plants *Selaginella moellendorffii* and *Selaginella uncinata*, respectively (Tsuji et al., 2007; Hecht et al., 2011). Like *C. subellipsoidea*, *Selaginella* species have a GC-bias in both their mitochondrial and plastid compartments (Smith, 2009). Plastid gene sequences of more than 100 *Selaginella* species from diverse regions revealed only one species without GC-biased ptDNA: the Chinese specimen *Selaginella sinensis* (Smith, 2009). Other lycophytes, including several *Isoetes* species, also have relatively high mtDNA GC contents (Malek and Knoop, 1998; Grewe et al., 2009). Analyses of *rbcL* genes suggests that some ferns from the genera *Cheiropleuria*, *Coniogramme*, *Cystopteris*, *Dryopteris*, and *Monachosorum* have a GC-bias in their ptDNA (Ebihara et al., 2010; de Groot et al., 2011).

### Animals and fungi

There are at least 25 species of fish with overall mtDNA GC contents >50%, such as the wolf herring *Chirocentrus dorab* (53%), the Pacific porthole fish *Diplophos taenia* (52%), and the beaked salmon *Gonorynchus greyi* (52%; Miya and Nishida, 2000; Saitoh et al., 2003; Ishiguro et al., 2005). Moreover, *cox1* nucleotide content analyses suggest that potentially hundreds, if not thousands, of other teleosts, from many different orders, have GC-biased mtDNA. Single-gene nucleotide content analyses have revealed various birds, frogs, and corals with GC-rich mitochondrial genomes (Kerr et al., 2007; Crawford et al., 2010). The European penduline tit *Remiz pendulinus*, the turtle frog *Myobatrachus gouldii*, and the zoanthid coral *Mesozoanthus fossii* all appear to have particularly high mtDNA GC contents. Among fungi, the pathogenic yeast *Candida subhashii* is the only species known to have GC-rich mtDNA (53%; Fricova et al., 2010).

### Diplonemids

One of the earliest discoveries of GC-rich mtDNA came from the *cox1* sequence of the euglenozoan *Diplonema papillatum*, a unicellular phagotrophic marine flagellate (Maslov et al., 1999). Further sequencing of mtDNA from this species has confirmed that its mitochondrial genome is enriched in G and C (∼55%; Vlcek et al., 2011). Other investigated members of the genus have AT-rich mtDNAs (Kiethega et al., 2011).

## Organelle Genome Architecture and GC Content

The available GC-rich organelle genomes (Figures 1 and 2) vary greatly in size, gene content, and coding density (Table 1). For instance, the mtDNA of *P. capuana* is small and compact (13 kb, >80% coding, and no introns; Smith and Lee, 2008) whereas that of *S. moellendorffii* is large and distended (250 kb, >80% non-coding, and 37 introns; Hecht et al., 2011). There are, however, several reoccurring architectural themes among GC-biased organelle genomes (Table 1).

In the mtDNAs of the chlorophyceans *L. culleus* and *P. capuana* and the yeast *C. subhashii*, a high GC content is partnered with a linear genome conformation and, for the latter two species, distinct telomeric structures (Smith and Lee, 2008; Borza et al., 2009; Fricova et al., 2010). GC-rich mtDNAs are sometimes fragmented into multiple chromosomes, as seen in *Oogamochlamys* algae and the euglenozoan *D. papillatum* (Borza et al., 2009; Vlcek et al., 2011); these same taxa, along with *P. capuana* and *S. moellendorffii*, also contain fragmented and/or trans-spliced mtDNA genes (Kiethega et al., 2011).

For some species, a high organelle GC content is associated with a small number of tRNA-coding regions: *P. capuana* and *S. moellendorffii* have the most reduced mitochondrial tRNA-coding suites observed from the Archaeplastida: 1 and no tRNAs, respectively. A low tRNA content is also found in the *Selaginella* plastid genome (Tsuji et al., 2007; Smith, 2009) and the mtDNAs of *D. papillatum* and zoanthid corals (Sinniger et al., 2007; Vlcek et al., 2011).

In certain cases, organelle genome GC richness is allied with high levels of post-transcriptional editing, particularly cytosine-to-uracil changes. Hundreds of C-to-U editing sites have been identified in the GC-biased mitochondrial and plastid genomes of *Selaginella* species (Tsuji et al., 2007; Smith, 2009; Hecht et al., 2011). And for land plants as a whole there is a positive relationship between organelle GC content and the abundance of C-to-U editing sites (Jobson and Qiu, 2008). In the GC-rich mtDNA of *D. papillatum*, some mitochondrial transcripts experience U-insertion-type RNA editing (Kiethega et al., 2011). Given that organelle RNA editing tends to be a uracil-enriching process, it may turn out that some GC-rich mtDNAs and ptDNAs, once all of their edited sites are uncovered, have AT-rich transcriptomes.

## What’s Causing Organelle Genome GC Enrichment?

Examining the distribution of GC among different regions within a genome, different genomes within a cell, and different species within a group can give insights into the forces that govern GC composition. The available GC-rich organelle DNAs come from an assortment of taxa belonging to disparate lineages (Figure 3). In some cases, the GC-bias is found in both the mitochondrial and plastid compartments of a species and in multiple species within a group, as observed for the spikemoss *Selaginella* and the trebouxiophyte *Coccomyxa* (Figure 3). In other examples, the GC-bias is restricted to either the mtDNA or ptDNA and/or is present in only a single species within the group, as seen for the green algae *Polytomella* and *Picocystis* (Figure 3). This variation in the presence and absence of GC-rich organelle DNA indicates that the processes biasing mitochondrial and plastid genomes in GC likely differ between lineages.

For many GC-rich organelle genomes, particularly those of green algae and the coral *Mesozoanthus fossii* (Figures 1 and 2), the concentration of GC is highest at silent sites, such as non-coding and synonymous sites (Table 1). This implies that in some organelle systems there is a non-adaptive underpinning to the GC-bias (Kimura, 1983). Two non-adaptive processes that can influence nucleotide landscape are biased mutation pressure and biased gene conversion. In most species, mtDNA and ptDNA mutation pressure seems to be skewed toward A and T (discussed above). Gene conversion, however, favors G and C in most genomes in which it has been studied (Mancera et al., 2008; Duret and Galtier, 2009; Muyle et al., 2011), with the exception of the tobacco ptDNA where it is AT biased (Khakhlova and Bock, 2006). Genomic regions with high rates of recombination undergo more gene conversion events than those with low recombination rates. In this context, it is noteworthy that some GC-rich organelle genomes are highly recombinogenic (Dieckmann and Gandy, 1987; Smith and Lee, 2008; Borza et al., 2009; Hecht et al., 2011), which may be a sign of a GC-biased conversion process. Moreover, in a variety of organelle genomes, including AT-rich ones, repeat elements (sequences that presumably undergo high levels of recombination) often have inflated GC contents (de Zamaroczy and Bernardi, 1986; Nedelcu and Lee, 1998). DNA methylation can also influence GC content – by promoting cytosine deamination events – but GC-rich mtDNAs and ptDNAs, like those of *S. moellendorffii*, do not have lower levels of methylation than those that are AT-rich (Zemach et al., 2010).

In other organelle DNAs, like those from land plants, the GC content is highest at functionally constrained sites, such as first and second codon positions (Table 1), suggesting that the GC-bias is the product of natural selection. Complicating this interpretation, however, is the fact that many of the cytosines residues at the non-silent sites from these taxa are post-transcriptionally edited to uracil (Jobson and Qiu, 2008; Smith, 2009; Hecht et al., 2011). Other adaptive hypotheses for a high GC composition include increased DNA thermo stability and UV tolerance. But these arguments seem implausible given that many GC-rich organelle DNAs come from species living in extremely cold habitats (e.g., *C. subellipsoidea* originates from Marble Point Antarctica) or environments with little UV light (e.g., the pathogenic yeast *C. subhashii*; Table 1).

For some species there is a correlation between lifestyle and organelle DNA GC content. Within the *Coccomyxa* genus, the three taxa known to have GC-rich organelle genomes are non-lichenized, free-living species, whereas all investigated symbiont *Coccomyxa* species have AT-rich organelle DNA (Smith et al., 2011). In the case of *Candida*, a parasitic lifestyle correlates with extreme organelle genome nucleotide compositions: the mtDNA of *C. subhashii* has one of the highest GC contents observed from the opisthokonts (Fricova et al., 2010) and that of its close relative *Candida castellii* is remarkably AT-rich (87%; Figure 1; Bouchier et al., 2009). The high mitochondrial GC contents of certain animals, such as frogs and fish (Figures 1 and 2), may be a reflection of them having low metabolic rates and consequently reduced mtDNA damage from oxygen free radicals (Martin, 1995).

Although data are limited, organelle DNA GC enrichment does not appear to be associated with nuclear DNA GC enrichment: *C. subellipsoidea* and *S. moellendorffii* have had their nuclear genomes completely sequenced (Banks et al., 2011; Blanc et al., 2012), revealing overall GC contents of ∼50%, which is unremarkable relative to the nuclear genomes of other green plants. However, the availability of these nuclear sequences will allow researchers to explore the full complement of nuclear-encoded mitochondrial- and plastid-targeted proteins, which should give insight into the biochemical and metabolic processes occurring within these organelles. Already, it has been revealed that the *C. subellipsoidea* nuclear genome lacks the plastid-targeted gene for the photosystem 1 (PSI) reaction center subunit N (*psaN*), which codes for a protein involved in the docking of plastocyanin. Interestingly, *psaN*-lacking strains of *Arabidopsis*, although maintaining a functional PSI complex, have reduced rates of electron transfer from plastocyanin to PSI (Haldrup et al., 1999). It is hypothesized that for *C. subellipsoidea* the unique loss of *psaN* may lead to reduced ROS formation (Blanc et al., 2012), which could help explain the high GC content of its organelle DNAs.

## Concluding Remarks

Organelle genomes are models for studying the evolution of genome size and structure (Nosek and Tomáska, 2003; Lynch et al., 2006). Now, with the discovery of GC-rich mtDNAs and ptDNAs, they have established themselves as excellent systems for exploring the origins of nucleotide landscape. The presence of GC-biased organelle DNA in key research lineages, like *Selaginella*, *Candida*, and Chlamydomonadalean algae, and the availability of complete organelle and nuclear genome sequences from these groups provide promising avenues for future studies on nucleotide composition. I predict that in the years to come GC-rich organelle DNAs will help further our understanding of nucleotide composition and its relationship with other aspects of genome architecture.

## Conflict of Interest Statement

The author declares that the research was conducted in the absence of any commercial or financial relationships that could be construed as a potential conflict of interest.
